# A study of diet in older community-dwelling adults in the UK during the COVID-19 pandemic: Findings from the Southampton Longitudinal Study of Ageing (SaLSA)

**DOI:** 10.3389/fnut.2022.988575

**Published:** 2023-01-13

**Authors:** Faidra Laskou, Gregorio Bevilacqua, Leo D. Westbury, Ilse Bloom, Pritti Aggarwal, Cyrus Cooper, Harnish P. Patel, Elaine Dennison

**Affiliations:** ^1^MRC Lifecourse Epidemiology Centre, University of Southampton, Southampton, United Kingdom; ^2^NIHR Southampton Biomedical Research Centre, University of Southampton and University Hospital Southampton NHS Foundation Trust, Southampton, United Kingdom; ^3^Living Well Partnership, Southampton, United Kingdom; ^4^Medicine for Older People, University Hospital Southampton, Southampton, United Kingdom

**Keywords:** diet, older adults, COVID-19, sarcopenia, frailty

## Abstract

**Introduction:**

Adequate nutrition is important for health in later life. Older adults are especially vulnerable to adverse outcomes following infection by COVID-19 and have commonly spent a disproportionate time within their own homes to reduce risk of infection. There are concerns that advice to shield may have led to malnutrition as older adults may modify daily routines including usual shopping habits. The aims of this study were to report self-reported pandemic-related changes in diet and examine lifestyle and medical correlates of these changes in older UK community-dwelling adults.

**Methods:**

We recruited 491 participants from the city of Southampton, UK. Participants completed a postal questionnaire in summer/autumn 2021, over a year after the first UK national lockdown was announced. The questionnaire ascertained demographic and lifestyle factors, in addition to number of comorbidities, nutrition risk scores, and presence of frailty. Associations between these participant characteristics in relation to self-reported changes in diet quality (lower, similar or higher when compared to before the first lockdown) were examined using ordinal logistic regression.

**Results:**

Median (lower quartile, upper quartile) age was 79.8 (77.0, 83.7) years. Overall, 11 (4.9%) men and 25 (9.4%) women had poorer diet quality compared to before the first UK lockdown. The following participant characteristics were associated with increased risk of being in a worse category for change in diet quality after adjustment for sex: lower educational attainment (*p* = 0.009); higher BMI (*p* < 0.001); higher DETERMINE (a malnutrition assessment) score (*p* = 0.004); higher SARC-F score (*p* = 0.013); and self-reported exhaustion in the previous week on at least 3 days (*p* = 0.002).

**Conclusions:**

Individuals at higher nutritional risk were identified as reporting increased risk of deterioration in diet quality during the pandemic. Further investigation of the factors leading to these changes, and an understanding of whether they are reversible will be important, especially for future pandemic management.

## 1. Introduction

Frailty is a common condition in late adulthood and is associated with very significant mortality and morbidity (1). Frailty is defined as an increased vulnerability to stressors across multiple bodily systems and sarcopenia is considered to be one of the key pathologies underpinning the frailty syndrome (1). Adequate nutrition is known to be associated with better musculoskeletal health in later life (1, 2). More specifically, sufficient intake of calcium, protein and other vitamins and minerals have been linked to better muscle health (3). Several epidemiological studies have explored the association of dietary habits or healthier diets with muscle health, and the association of individual foods and nutrients with fracture, falls and frailty (4–7).

The emergence of the coronavirus disease (COVID-19), declared a pandemic by the World Health Organization in March 2020, and subsequent restrictive measures introduced in many countries to reduce the transmission of the disease, may have negatively impacted daily routines and lifestyle. This is especially likely among older adults, who are at greatest risk of complications from infection by the COVID-19 virus (8, 9). Potential factors associated with changes in diet quality remain controversial.

We hypothesized that the COVID-19 pandemic may have a differential impact on diet of older adults in the UK dependent on other sociodemographic and medical factors. We therefore aimed to report pandemic-related changes in diet and examine correlates of changes in diet quality in a newly established community-dwelling older adult cohort in the UK, to consider who might be at greatest risk of malnutrition.

## 2. Methods

### 2.1. Southampton longitudinal study of ageing (SaLSA)

In July 2021, we identified all patients who were registered at a large General Practice within Southampton, UK over the age of 75 (10). Eligibility to participate in the study was decided by their primary care physician. All participants were >75 years of age at the time of recruitment. We did not approach patients who their primary care physician reported as having any of the following problems; safeguarding issues, patients with mental health and capacity issues, patients with dementia or who were unable to provide consent, patients with learning disabilities, patients who were end of life, patients who were permanently bed bound, and patients in residential or nursing homes. Of 2,523 registered patients over the age of 75 years at the practice, 1993 (79%) were eligible to participate in the study based on the criteria above and 516 complete questionnaires were received. This study was conducted according to the guidelines of the Declaration of Helsinki, and approved by the Research Ethics Committee (REC) Health Regulator Authority (HRA) (REC reference 21/SC/0036, 17 March 2021).

### 2.2. Ascertainment of participant characteristics

All participant information was self-reported using postal questionnaires. Sociodemographic factors included: age; sex; ethnicity; educational qualifications achieved; living arrangements; and current marital status. Participants were asked to indicate if they had any of the following qualifications: O Levels/GCSEs; A Levels; vocational training certificates; university degree(s) or Higher National Diploma; or higher professional qualifications. Participants were asked to select one of the following options for their living arrangement: their own property; rented accommodation; residential home; nursing home; or “other.” Self-reported height and weight were used to derive BMI (kg/m^2^). Information on health behaviors such as smoking, alcohol consumption, and information on malnutrition risk using the DETERMINE tool (11) were also ascertained. In brief, DETERMINE is a tool designed by the American Academy of Family Physicians, the National Council on the Aging and others as part of the Nutrition Screening Initiative which can be used by professionals working with older people to assess their risk for poor nutritional status or malnutrition. Change in the following aspects of lifestyle compared to before the first UK lockdown (23/03/2020) were reported: smoking, alcohol consumption, diet quality (reported as “healthiness of diet”), amount of food consumed, physical activity, sleep, and social contact. The questions used were taken from the Wellcome COVID questionnaire, which was designed to enable researchers to collect COVID related data using the same validated questions used or adopted by other cohorts (accessed at https://www.bristol.ac.uk/alspac/researchers/wellcome-covid-19/) (12). For example, to ascertain change in diet quality, participants were asked to select one of the following options to describe the current healthiness of their diet compared to before the first UK lockdown: “less healthy than before”; “about the same healthiness as before”; or “more healthy than before.”

Comorbidities were ascertained by asking participants whether they were ever diagnosed by a doctor with any of the following conditions: heart attack or angina; stroke or transient ischaemic attack; hypertension; diabetes; asthma, bronchitis, emphysema, or chronic obstructive pulmonary disease (COPD); depression; osteoporosis; anxiety; memory problems or dementia; Parkinson's disease; osteoarthritis or degenerative joint disease; rheumatoid/inflammatory arthritis; cancer; or high cholesterol. The resulting number of comorbidities reported was used as a marker of morbidity. The Strength, Ambulation, Rising from a chair, Stair climbing and history of Falling (SARC-F) screening tool for sarcopenia (13) and the following Fried frailty questions were also included (14): “In the last year, have you lost more than 10 pounds unintentionally?” and “How often in the last week did the following apply?” “I felt that everything I did was an effort” or “I could not get going”.

### 2.3. Statistical methods

Summary statistics were used to describe baseline participant characteristics and changes in the following aspects of lifestyle compared to before the first UK lockdown: smoking, alcohol consumption, diet quality, amount of food consumed, physical activity, sleep and social contact. Sex-adjusted ordinal logistic regression was used to examine risk of being in a worse category for change in diet quality (compared to before the first UK lockdown) according to various participant characteristics; sex-interaction effects were not statistically significant. The outcome, change in diet quality, was coded as follows: 1: higher than before; 2: about the same as before; 3: lower than before. Therefore, examples of a worse category for change in diet quality would be “lower than before” as opposed to “about the same” or “about the same” as opposed to “higher than before.” Participant characteristics considered as exposures included: sociodemographic factors, BMI, current health behaviors, number of comorbidities, SARC-F score, and Fried frailty components (unintentional weight loss and self-reported exhaustion). Cross-tabulations of pandemic-related changes in diet quality and physical activity were examined using chi-squared tests and Fisher's exact tests, as appropriate.

All analyses were conducted using Stata (StataCorp, College Station, TX, USA), release 17.0; *p* < 0.05 was regarded as statistically significant. The analysis sample comprised the 491 participants who had data on self-reported change in diet quality since before the first UK lockdown.

## 3. Results

### 3.1. Descriptive statistics

The participant characteristics of the analysis sample at baseline are presented in Table 1. Median (lower quartile, upper quartile) age was 79.8 (77.0, 83.7) years; 482 (99.0%) were of white ethnicity; 420 (87.7%) lived in their own property; and 260 (53.4%) were in a current relationship (married/civil partnership/cohabiting).

**Table 1:** Baseline participant characteristics.

**Participant characteristic**	**Mean (SD), median (lower quartile, upper quartile) or** ***n*** **(%)**	
	**Men (*****n*** = **225)**	**Women (*****n*** = **266)**
Age (years)	79.7 (76.7, 83.3)	80.1 (77.2, 84.4)
Education (University degree / HND / Higher professional qualifications)	55 (24.4%)	41 (15.4%)
**Living arrangement**		
Own property	191 (87.2%)	229 (88.1%)
Rented accommodation	24 (11.0%)	24 (9.2%)
Residential home	2 (0.9%)	0 (0.0%)
Nursing home	0 (0.0%)	0 (0.0%)
Other	2 (0.9%)	7 (2.7%)
**Current marital status**		
Single	10 (4.5%)	9 (3.4%)
Married or civil partnership	146 (65.8%)	100 (37.7%)
Divorced or separated	22 (9.9%)	22 (8.3%)
Widowed	35 (15.8%)	129 (48.7%)
Cohabiting	9 (4.1%)	5 (1.9%)
Self-reported height (cm)	174.5 (7.6)	160.6 (6.0)
Self-reported weight (kg)	79.4 (11.5)	68.0 (13.6)
BMI (kg/m^2^)	26.0 (3.5)	26.4 (5.2)
Ever smoked regularly	135 (60.5%)	95 (36.0%)
Currently drink alcohol	191 (84.9%)	188 (70.9%)
DETERMINE score	3.0 (1.0, 5.0)	3.0 (1.0, 4.0)
Number of comorbidities^+^	2.0 (1.0, 3.0)	2.0 (1.0, 3.0)
SARC-F score	0.0 (0.0, 2.0)	2.0 (1.0, 3.0)
Lost more than 10 pounds unintentionally in past year	22 (9.8%)	19 (7.3%)
**Self-reported exhaustion in the past week** ^*^		
Rarely or none of the time (<1 day)	116 (52.0%)	115 (44.4%)
Some or a little of the time (1–2 days)	66 (29.6%)	76 (29.3%)
A moderate amount of time (3–4 days)	22 (9.9%)	32 (12.4%)
Most of the time (>4 days)	19 (8.5%)	36 (13.9%)

HND: Higher National Diploma.

BMI was calculated from self-reported height and weight.

DETERMINE: DETERMINE Your Nutritional Health Checklist.

+ Number of the following doctor-diagnosed conditions: heart attack or angina; stroke or transient ischaemic attack; hypertension; type 1 or 2 diabetes; asthma, bronchitis, emphysema, or chronic obstructive pulmonary disease (COPD); depression; osteoporosis; anxiety; memory problems or dementia; Parkinson's disease; osteoarthritis or degenerative joint disease; rheumatoid/inflammatory arthritis; cancer; or high cholesterol.

SARC-F: Strength, Ambulation, Rising from a chair, Stair climbing and history of Falling screening test for sarcopenia.

* How often in the last week did the following apply? “I felt that everything I did was an effort” or “I could not get going”.

Changes in lifestyle compared to before the first UK lockdown are presented in Table 2. A greater proportion of participants consumed less alcohol than before (11.6% men, 9.8% women) compared to those that consumed more than before (3.7% men, 3.3% women); over 99% of men and women did not smoke or reported no change in their smoking behavior. Most participants reported no change in diet quality (88.9% men, 85.7% women) with smaller numbers reporting declines in diet quality (4.9% men, 9.4% women) and increases in diet quality (6.2% men, 4.9% women); a similar pattern was observed for amount of food consumed. Considerably more participants reported declines in physical activity, sleep quality and social contact in comparison to the number who reported increases. For example, 36.0% of men and 42.8% of women reported declines in physical activity whereas only 5.8% of men and 6.4% of women reported increases. Statistically significant sex-differences in these lifestyle changes were only observed for alcohol consumption (*p* = 0.001).

**Table 2:** Changes in aspects of lifestyle compared to before the first UK lockdown (23/03/2020).

**Participant characteristic**	***N* (%)**		*P*-value for sex-difference^*^	**Effect size (Cramér's V) for sex-difference**
	**Men (*****n*** = **225)**	**Women (*****n*** = **266)**		
**Smoking**			0.479	0.060
More than before	0 (0.0%)	0 (0.0%)		
About the same	5 (2.2%)	3 (1.1%)		
Less than before	0 (0.0%)	1 (0.4%)		
Does not smoke	219 (97.8%)	262 (98.5%)		
**Alcohol**			0.001	0.185
More than before	8 (3.7%)	8 (3.3%)		
About the same	149 (69.0%)	135 (55.3%)		
Less than before	25 (11.6%)	24 (9.8%)		
Does not drink alcohol	34 (15.7%)	77 (31.6%)		
**Diet quality**			0.141	0.089
Lower than before	11 (4.9%)	25 (9.4%)		
About the same	200 (88.9%)	228 (85.7%)		
Higher than before	14 (6.2%)	13 (4.9%)		
**Food consumption**			0.388	0.062
Less than before	27 (12.1%)	27 (10.2%)		
About the same	187 (83.5%)	219 (82.6%)		
More than before	10 (4.5%)	19 (7.2%)		
**Physical activity**			0.252	0.075
Less than before	81 (36.0%)	113 (42.8%)		
About the same	131 (58.2%)	134 (50.8%)		
More than before	13 (5.8%)	17 (6.4%)		
**Sleep**			0.558	0.049
Worse than before	45 (20.0%)	63 (23.9%)		
About the same	174 (77.3%)	193 (73.1%)		
Better than before	6 (2.7%)	8 (3.0%)		
**Social contact**			0.535	0.056
Less than before	156 (69.3%)	176 (67.2%)		
About the same	68 (30.2%)	82 (31.3%)		
More than before	1 (0.4%)	4 (1.5%)		

* P-values for sex-differences were derived from chi-squared tests; Fisher's exact tests were used for smoking and social contact due to the low expected frequencies in some of the cells.

### 3.2. Predictors of change in diet quality compared to before the first UK lockdown

Cross-sectional associations between participant characteristics and risk of being in a worse category for change in diet quality (compared to before the first UK lockdown) are presented in Table 3. The following participant characteristics were associated with increased risk of being in a worse category for change in diet quality after adjustment for sex: lower educational attainment (*p* = 0.009); higher BMI (*p* < 0.001); higher nutrition risk score (*p* = 0.004); higher SARC-F score (*p* = 0.013); and self-reported exhaustion in the previous week on at least 3 days (*p* = 0.002). For example, after adjustment for sex, the odds ratio (95% CI) for being in a worse category for change in diet quality was 1.13 (1.06, 1.19) per unit increase in BMI and 2.76 (1.47, 5.18) for those who reported exhaustion in the previous week on at least 3 days compared to those who did not.

**Table 3:** Sex-adjusted odds ratios for being in a worse category for change in diet quality compared to before the first UK lockdown according to participant characteristics.

**Participant characteristic**	**Odds ratio (95% CI)**	***P*-value**	**McFadden pseudo R^2^**
Age (years)	0.99 (0.94, 1.05)	0.765	0.0074
Education (degree/HND/professional qualifications vs. lower)	0.40 (0.20, 0.79)	0.009	0.0222
Living arrangement (own property vs. not)	1.19 (0.52, 2.71)	0.679	0.0080
Marital status (married/civil partnership/cohabiting vs. not)	0.89 (0.50, 1.56)	0.678	0.0081
BMI (kg/m^2^)	1.13 (1.06, 1.19)	<0.001	0.0444
Ever smoked regularly	0.58 (0.33, 1.03)	0.062	0.0143
Currently drink alcohol	1.36 (0.71, 2.60)	0.347	0.0091
DETERMINE score	1.16 (1.05, 1.29)	0.004	0.0243
Number of comorbidities	1.07 (0.91, 1.26)	0.435	0.0085
SARC-F score	1.19 (1.04, 1.36)	0.013	0.0200
Lost more than 10 pounds unintentionally in past year	1.44 (0.55, 3.76)	0.458	0.0090
Self-reported exhaustion in past week (≥3 days vs. <3 days)	2.76 (1.47, 5.18)	0.002	0.0290

Odds ratios were estimated using sex-adjusted ordinal logistic regression models.

### 3.3. Association between change in diet quality and change in physical activity

Changes in diet quality and physical activity compared to before the first UK lockdown were correlated among men (*p* = 0.002) and women (*p* = 0.007). For example, among men who did not report declines in physical activity, only 1.4% reported declines in diet quality, compared to 11.1% among those who did report declines in physical activity; corresponding figures among women were 5.3% and 15.0%, respectively (Table 4).

**Table 4:** Cross tabulations of declines in diet quality and physical activity since before the first UK lockdown among men and women.

			**Decline in physical activity**		
			**No**	**Yes**	**Total**
**Men**	Decline in diet quality	No	142 (98.6%)	72 (88.9%)	214 (95.1%)
		Yes	2 (1.4%)	9 (11.1%)	11 (4.9%)
		Total	144 (100%)	81 (100%)	225 (100%)
**Women**	Decline in diet quality	No	143 (94.7%)	96 (85.0%)	239 (90.5%)
		Yes	8 (5.3%)	17 (15.0%)	25 (9.5%)
		Total	151 (100%)	113 (100%)	264 (100%)

Column percentages are shown among men and women separately.

Correlations between declines in diet quality and physical activity were statistically significant among men (p = 0.002) and women (p = 0.007).

## 4. Discussion

This study has described changes in lifestyle factors and examined correlates of change in diet quality compared to before the first UK national lockdown in March 2020. Although self-reported changes in alcohol consumption and smoking were minimal, considerably more participants reported declines in physical activity, sleep quality and social contact in comparison to those who reported increases. Most participants reported no change in diet quality with small proportions reporting declines or increases in diet quality. However, the following were associated with increased risk of being in a worse category for change in diet quality after adjustment for sex: lower educational attainment; higher BMI; higher DETERMINE score; higher SARC-F score; and self-reported exhaustion. This suggests that there is a small subset of older community-dwelling adults whose diet deteriorated over the first year of the COVID-19 pandemic. Worryingly, this included individuals with a higher DETERMINE score, a score used by health professionals to identify older adults at risk of malnutrition and/or higher SARC-F score, where a cut-off of 4 or higher is suggestive of sarcopenia and poor outcomes.

Our results are in accord with previous work where in a study of Dutch adults, younger generations were more likely, when compared to older generations, to be influenced by COVID-19 lockdowns and to change their eating behaviors (15). This observation was also supported by a recent systematic review looking into the impact of the COVID-19 pandemic on diet where 6 out of 10 studies examined observed no significant changes in dietary habits of older adults (16). However, the included studies were heterogenous in findings, and did not consider correlates of dietary decline or associated health outcomes. For example, in 2 studies, no more than 50% of participants were found not to have altered their dietary habits (17, 18).

In other work, older participants tended to be less likely to report changes in food behaviors, overall diet healthfulness, and food security compared to younger participants in the International Food Policy Study conducted across 5 countries (19). Other researchers have reported that age was negatively associated with healthy dietary change (20, 21) and increased intake of junk food (22). Constant et al. found that individuals aged 40–60 and 60+ years were around 20% less likely to have made a “positive” lifestyle change in eating habits during the lockdown period in France (23). Similar findings were reported in a recent study by Bevilacqua et al. of participants from two UK cohorts of community-dwelling older adults: during the first months of the pandemic, individuals from the younger cohort (median age 65 years) reported more negative changes to their diets when compared to those from the older cohort (median age 84 years) (24). In addition, Bevilacqua et al. reported that younger women were more likely to have increased their food intake and reduced the quality of their diet compared to men (24), a tendency that we also notice in our sample population, with more women than men reporting both a decrease in diet quality (9.4% vs. 4.9%) and an increase in food intake (7.2% vs. 4.5%); however, in our study this difference was not statistically significant. Quarantine due to the COVID-19 pandemic had a negative impact on physical and mental health as well as on lifestyle in older adults from five Central American countries; a greater effect was reported on having a balanced diet, frequency of falls, and functional ability amongst others (25). Luo et al. also report that older individuals were more likely to change dietary habits during the pandemic (26).

Differences in socioeconomic status and food availability, especially during the early phases of the pandemic, may account for some of the differences observed between studies. Energy-dense foods with high sugar and fat may be cheaper and more palatable, with access different in different groups of adults in different countries (27). Lower educational attainment was associated with an increased risk of being in a worse category for change in diet quality in our cohort. Participants with higher adherence to a Mediterranean diet during the COVID-19 pandemic were more likely to have a higher education level in a study in Spain; education level was related to following a Mediterranean diet while advised to stay at home during early pandemic restrictions (28). Higher educational level has been commonly associated with higher socioeconomic status which is often related to better diet quality (27). Finally higher education levels predict an increase in healthier dietary patterns in non-COVID related studies (29).

While dietary change during the pandemic has been studied by other researchers (29, 30), far fewer have considered much older adults. The dietary habits of older people living with frailty may have been more strongly affected by social isolation during the COVID-19 pandemic than those who were not frail as reported in one study. In addition, reduced food shopping times during social isolation was significantly higher among frail individuals while frail older adults consumed less high-quality proteins but more protein rich foods and vegetables which might indicate an attempt to improve dietary habits (30). These observations accord with our work where self-reported exhaustion, one of the elements of the Fried Frailty criteria, was associated with an increased risk of being in a worse category for change in diet quality. It is of course possible that the deterioration reported by some older adults was unrelated to the COVID-19 pandemic. While this is possible, recent qualitative work in another older UK cohort would suggest that although causality cannot be established, the pandemic has impacted the way older adults access shops (31).

This study has some limitations. First, all information was ascertained through self-reported questionnaires. Second, recall bias may have occurred as participants reported changes in lifestyle factors since the UK national lockdown instead of reporting current lifestyle behaviors at two separate time points before and after the lockdown. Third, investigating the impact of having COVID-19 or experiencing COVID-19 related symptoms on changes in lifestyle factors was not possible as only a very small proportion of participants had a confirmed COVID-19 infection. Furthermore, detailed dietary data were not available. In our study, 1,993 patients were invited to participate in the study by postal invitation which led to the return of 516 complete questionnaires (26% response rate). However, a previous study which tested the feasibility of setting up a UK sarcopenia registry only achieved a 12% response rate when potential participants aged 65 and over were approached *via* mailshots from local primary care practices (32). This suggests that our response rate was not unusually low, given that we were contacting participants from the general population. Fourth, participants completed questionnaires from July 2021 until April 2022; the COVID-19 UK Government guidelines and restrictions changed during this time so the reference point for comparing their lifestyles from before the first UK lockdown would have differed between participants. Fifth, the fairly small sample size used for analysis may have limited the power to detect statistically significant differences in declines in diet quality according to the various participant characteristics considered. Finally, as in all cohort studies, participants who consented to be included are likely to have been healthier than those who refused which may have limited the generalisability of findings. This is supported by the fact that 87.7% of participants lived in their own property and 53.4% were in a current relationship. However, as our analyses were internal, substantial bias should only have been introduced if the associations examined differed markedly between those who participated in the study and those who did not; this seems unlikely. To confirm this, we assessed demographic characteristics of our population against national survey data for England (accessed at https://digital.nhs.uk/data-and-information/publications/statistical/health-survey-for-england); prevalence of current smoking in our analysis sample (men 3%, women 2%) was slightly lower than reported among participants aged 75 years and older from the nationally representative 2019 Health Survey for England (men 6%, women 6%). However, these are fairly similar, given the variability expected from the reasonably small SaLSA sample and also the Health Survey for England which reported a 3% current smoking prevalence among men aged 75 years and older in 2018. However, mean BMI in our analysis sample (men 26.0, women 26.4) was slightly lower than reported among participants aged 75 years and older from the 2019 Health Survey for England (men 27.6, women 27.7), suggesting that our cohort may indeed be slightly healthier than national averages. Finally, although all data collected were obtained through self-administered questionnaires, we used validated tools wherever possible.

## 5. Conclusion

Older individuals are a particularly vulnerable population for COVID-19 infection and were commonly advised to shield to reduce exposure to the virus, which may have impacted access to usual food choices. We have found that greater nutritional risk was associated with being in a worse category for change in diet quality, highlighting the need to consider this when providing support for shopping and nutritional guidance to older adults in any future pandemics and should be considered when evaluating older adults as we emerge from the current pandemic.

## Data availability statement

The datasets presented in this article are not readily available because a steering committee will be set up to manage data access requests. Requests to access the datasets should be directed to faidra.laskou@soton.ac.uk.

## Ethics statement

The studies involving human participants were reviewed and approved by the Research Ethics Committee (REC) Health Regulator Authority (HRA) (REC reference 21/SC/0036, 17/03/2021). The patients/participants provided their written informed consent to participate in this study.

## Author contributions

FL produced the first draft of the manuscript. LW conducted the statistical analyses. GB, IB, and HP made substantial additions to the manuscript. ED and CC designed the study and revised the manuscript. All authors made substantial contributions to the manuscript and approved the final version.
